# Hardness and Indentation Fracture Toughness of Slip Cast Alumina and Alumina-Zirconia Ceramics

**DOI:** 10.3390/ma13010122

**Published:** 2019-12-26

**Authors:** Irena Žmak, Danko Ćorić, Vilko Mandić, Lidija Ćurković

**Affiliations:** 1 Department of Materials, Faculty of Mechanical Engineering and Naval Architecture, University of Zagreb, HR-10000 Zagreb, Croatia; irena.zmak@fsb.hr (I.Ž.); lidija.curkovic@fsb.hr (L.Ć.); 2 Department of Inorganic Chemical Technology and Non-Metals, Faculty of Chemical Engineering and Technology, University of Zagreb, Marulićev trg 20, 10000 Zagreb, Croatia; vmandic@fkit.hr

**Keywords:** alumina, zirconia, slip casting, Vickers hardness, fracture toughness

## Abstract

Alumina (Al_2_O_3_) and zirconia (ZrO_2_) have good overall properties and thus are widely used oxide technical ceramics. The biggest drawback of Al_2_O_3_ is its low fracture toughness. In contrast, ZrO_2_ is relatively tough, but is also much more expensive. In order to improve the alumina toughness, composite ceramics are being developed. Slip casting technology has economic advantages over the conventional hot isostatic pressure technology, but problems may arise when preparing stable highly-concentrated suspensions (slip) for filling the mold. The purpose of this study is to prepare aqueous suspensions using 70 wt. % α-Al_2_O_3_, with 0, 1, 5 and 10 wt. % of added *t*-ZrO_2_. Suspensions were electrosterically stabilized using the ammonium salt of polymethylacrylic acid, an alkali-free anionic polyelectrolyte dispersant. Also, magnesium oxide in form of magnesium aluminate spinel (MgAl_2_O_4_) was used to inhibit the abnormal alumina grain growth during the sintering process. Minimum viscosities were used as stability estimators, where an increase in ZrO_2_ content required adding more dispersant. After sintering, the Vickers indentation test was used to determine the hardness and the indentation fracture toughness from the measurement of the crack length. Also, the brittleness index (*B*_i_, μm^−1/2^) was calculated from values of Vickers hardness and the Vickers indentation fracture toughness. It was found that with increasing ZrO_2_ content the fracture toughness increased, while the hardness as well as the brittleness index decreased. Zirconia loading reduces the crystallite sizes of alumina, as confirmed by the X-ray diffraction analysis. SEM/EDS analysis showed that ZrO_2_ grains are distributed in the Al_2_O_3_ matrix, forming some agglomerates of ZrO_2_ and some pores, with ZrO_2_ having a smaller grain size than Al_2_O_3_.

## 1. Introduction

Aluminum oxide (Al_2_O_3_) is the most important technical material of the oxide ceramics group, suitable for various applications in the electrical, electronic, chemical and medical industries. Densely sintered Al_2_O_3_ ceramic is characterized by low fracture toughness and high hardness, temperature stability, good wear resistance, corrosion resistance at elevated temperatures and excellent biocompatibility. A major demerit of aluminum oxide is its pronounced brittleness, that is, its relatively low fracture toughness which is 4–6 MPa m^½^. After the start of cracking, its propagation does not stop by plastic deformation, but continues until fracture. This phenomenon is usually caused by individual defects on the surface or very close to the surface of the material, since it is the site of greatest stress [1,2,3].

Pure zirconium oxide (ZrO_2_) also belongs in the oxide ceramics group. It has almost ideal properties: high fracture toughness (up to 15 MPa m^½^), high flexural and tensile strength, high wear resistance and corrosion resistance, low thermal conductivity, good thermal shock resistance, resistance to high temperatures and excellent biocompatibility [3,4].

Pure ZrO_2_ occurs in three polymorphic modifications: monoclinal, *m* (from room temperature up to 1170 °C), tetragonal, *t* (1170 °C–2370 °C) and cubical, *c* (from 2370 °C to melting point). During cooling, the transition from tetragonal to monoclinic phase takes place at a temperature of about 100 °C below 1170 °C, whereby the volume increases by 3–5%. Because of the stress resulting from the phase transformation, while cooling from sintering temperature (1500–1700 °C), cracking occurs in the final product. To avoid phase transitions and thus cracking, ZrO_2_ is stabilized by the addition of metal oxides (CaO, CeO_2_, MgO, Y_2_O_3_) [4].

By mixing ceramic powders in the initial phase of forming it is possible to produce Al_2_O_3_–ZrO_2_ composite ceramics, which exhibit better properties compared to monolithic Al_2_O_3_, respectively ZrO_2_ ceramics. Since the addition of ZrO_2_ increases the fracture toughness of Al_2_O_3_ ceramics, such composite ceramics are referred to as zirconia toughened alumina–ZTA in literature [5,6]. Stress that occurs under increased strain conditions can cause cracks in the ceramic material. In case of Al_2_O_3_–ZrO_2_ ceramic, crack formation is mitigated on behalf of a phase transition. Namely, ZrO_2_ grains that are found in the cracking zone undergo phase transformation from tetragonal into monoclinic phase, where the corresponding volume change facilitates closure of the cracks and prevents further propagation. These favorable mechanical and tribological properties of Al_2_O_3_–ZrO_2_ composite ceramics make it suitable for use in many areas, including cutting tools and implants. Al_2_O_3_–ZrO_2_ is a perspective biomaterial also, since, apart from biocompatibility and mechanical properties, it also meets the aesthetic criteria [5,6,7,8,9].

Contemporary trends in material development focus on improving the properties of existing materials as well as developing new ones. In order to lower their production costs and make them more environmentally friendly, the interest in the production of technical ceramics by slip casting has increased in the last few years. This technology is inexpensive, simple, fast, environmentally friendly and flexible. It enables production of ceramic products of different sizes and form complexities but requires an adequate understanding of colloidal solutions in order to optimize process parameters for the final ceramic product to have the required mechanical and other properties [10].

The properties of ceramic products obtained by slip casting depend on particle size of the ceramic powders and their proportion in the suspension. Generally, smaller particles and higher suspension concentration ultimately result in better properties. However, high suspension concentrations and particle diameter less than 1 mm cause enhanced interactions between particles, which significantly increases viscosity and makes it difficult to cast the suspension into a gypsum mold [3,10,11,12].

Many studies are focused on observing the influence of certain kinds and amounts of additives on the viscosity of ceramic suspensions and consequent properties of the final ceramic product [13,14,15,16,17,18].

**Figure 1:**
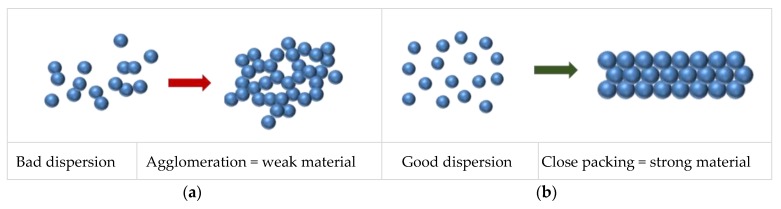
The influence of particle dispersion on properties on sintered ceramics: (**a**) bad dispersion and (**b**) good dispersion

For reasons mentioned above, highly concentrated suspensions are stabilized by addition of various additives (dispersants). The difference between the ceramic product obtained by casting a stable and an unstable suspension is best illustrated in Figure 1. If an unstable suspension containing irregular aggregates of particles (agglomerates) is poured into a mold, the particles are arranged into an irregular structure when dry (draining of water into mold walls), leaving cavities (voids) and irregularities in the microstructure of the raw material (Figure 1a). By sintering, these irregularities are further enhanced, resulting in a ceramic product of unsatisfactory properties. If the suspension is stable, by drying the particles become densely arranged, which after sintering gives a ceramic product of the appropriate mechanical and other properties (Figure 1b).

Agglomeration and sedimentation should be prevented by enhancing the rejection forces between particles of the ceramic powder. These forces must be strong enough to overcome the attractive, van der Waals force [7].

These interaction can be controlled with chemical additives in three different ways: electrostatic, steric, and electrosteric (a combination of the first two) stabilization. The influence of additives (different dispersants) on the ceramic suspension stability has been extensively researched [13,14,15,16,17,18,19].

Some of the dispersants which have been used as stabilizing agents for preparation of stable aqueous alumina suspension include ammonium polymethacrylate (“Darvan C”) [19,20,21], 4,5-dihydroxy-1,3-benzenedisulfonic acid disodium salt (“Tiron”) [21,22,23], triammonium salt of aurintricarboxylic acid (“Aluminon”) [21], (Darvan C-N) [23], sodium pyrophosphate, diammonium hydrogen citrate [24], citric acid [23], ammonium polyacrylate (“Seruna D-305”) [25], carbonic acid salt (“Dolapix CE 64”) [20,26,27], polycarbonic acid salt (“Dolapix PC 33”) and carbonic acid ester (“Dolapix ET 85”) [27].

The stability of the suspension is tested by sedimentation tests, by measuring the zeta potential and the particle size in the suspension and by determining rheological parameters [6,8]. The goal of the presented study was the preparation of stable Al_2_O_3_ and Al_2_O_3_-ZrO_2_ suspension by electrosteric stabilization suitable for slip casting. In addition, the main interest of this research is the effect of ZrO_2_ content (0, 1, 5 and 10 wt. %) on the microstructure and mechanical properties (hardness, fracture toughness and brittleness index) of sintered Al_2_O_3_-ZrO_2_ composite ceramics.

## 2. Materials and Methods

### 2.1. Ceramic Powder and Reagents

Samples of monolithic Al_2_O_3_ and composite Al_2_O_3_-ZrO_2_ were prepared by the slip casting technique. For preparation of highly concentrated aqueous suspensions (slips) following components were used:

- High-purity Al_2_O_3_, with average particle size of 300–400 nm (Alcan Chemicals, Stamford, CT, USA)
- High-purity ZrO_2_ stabilized with 3 mol % of yttria (Y_2_O_3_), with average particle size of 25 nm (SkySpring Nanomaterials Inc., Houston, TX, USA)
- An alkali-free anionic polyelectrolyte dispersant Dolapix CE 64 (Zschimmer & Schwarz GmbH &Co KG Chemische Fabriken, Lahnstein, Germany)–70 wt. % aqueous solution of the ammonium salt of polymethacrylic acid (PMAA-NH_4_)
- Magnesium oxide added as magnesium aluminate spinel (MgAl_2_O_4_) made by Alfa Aesar, Haverhill, MA, USA was used to inhibit the abnormal alumina grain growth during the sintering process [22]. Magnesium spinel is segregated on the grain boundaries of alumina grains and reducing the mobility of the grain boundaries
- Deionized water

Chemical composition of the Al_2_O_3_ and ZrO_2_ powders, according to the manufacturer’s data, is given in Table 1 and Table 2, respectively.

In the present study, powder X-ray diffraction (PXRD) analysis was used in order to determine phase compositions of the raw Al_2_O_3_ and ZrO_2_ powder and heat treated Al_2_O_3_-ZrO_2_ composites at 1650 °C. The device used was Shimadzu XRD6000 (Shimadzu Corporation, Kyoto, Japan) X-ray diffractometer with CuKα radiation. The fixed step scans were collected in the 2θ range 20–60° with steps of 0.02° 2θ and counting time 0.6 s under accelerating voltage of 40 kV and current of 30 mA.

### 2.2. Suspension Preparation and Characterization

Four groups of alumina-zirconia aqueous suspensions were prepared. All suspensions contained 70 wt. % of dry ceramic powder and 30 wt. % of deionized water. The dry powder composition was as follows:

- 100 wt. % Al_2_O_3_
- 99 wt. % Al_2_O_3_ and 1 wt. % of ZrO_2_
- 95 wt. % Al_2_O_3_ and 5 wt. % of ZrO_2_
- 90 wt. % Al_2_O_3_ and 10 wt. % of ZrO_2_.

**Figure 2:**
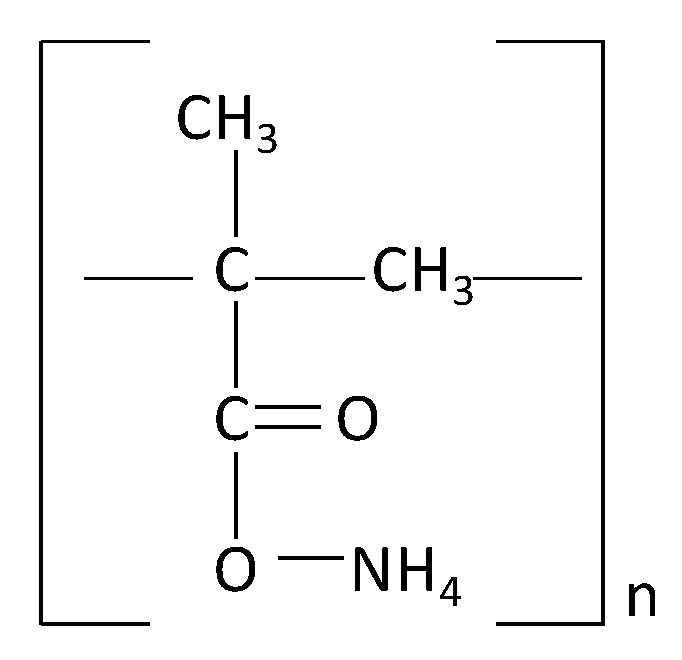
The molecule structure of dispersant Dolapix CE 64.

DOLAPIX CE 64 dispersant was used for the electrosteric stabilization of all ceramic suspensions. The structure of the functional group of the dispersant is shown in Figure 2.

The optimal amount of the dispersant was determined for each suspension separately. The composition of the different suspensions for determining the optimal amount of DOLAPIX CE 64 are given in Table 3. The optimal amount of dispersant was determined by measuring the apparent viscosity as a function of the amount of dispersant added. In previous research, it was found that addition of magnesium aluminate spinel (MgAl_2_O_4_) does not affect the apparent viscosity of the high concentrate alumina suspension [22]. The viscosity is at minimum value when the dispersion of ceramic particles is optimal.

For determining the apparent viscosity all suspensions were prepared by adding deionized water containing dissolved DOLAPIX CE 64 into the grinding jar of a planetary ball mill, after which ceramic powders were added. The grinding jar and ten balls used for homogenization were made of alumina ceramics in order to prevent the contamination of suspensions. Each of the prepared suspensions were homogenized for 90 min at a rate of 300 rpm in the planetary ball mill (PM 100, Retsch, Haan, Germany). After mixing, the ceramic balls were separated from the suspensions. Prior to the apparent viscosity measurement and forming of the green bodies, suspensions were ultrasonically treated in the ultrasonic bath BRANSONIC 220 (Branson Ultrasonics Corp., Danbury, CT, USA) with 50 kHz frequency and power of 120 W to remove trapped air bubbles and agglomerates, as well as tempered at 25 ± 1 °C with the assistance of the thermostatic bath Lauda Eco RE 415 (LAUDA-Brinkmann, Delran, NJ, USA).

The apparent viscosity of each suspension was determined by means of the rotational viscometer DV-III Ultra (Brookfield Engineering Laboratories, Middleboro, MA, USA) with small sample chamber and SC4-18 spindle. Viscosity was determined at the shear rate of 50 s^−1^, which is the exact shear rate of gravity slip casting.

After completing the rheological measurements and finding the optimum amount of the dispersant, sedimentation tests were performed. Four groups of suspensions were additionally prepared with the optimum amount of dispersant. The pH-values of these prepared suspensions were determined on the FE20/EL20 pH meter (error range 0.01) manufactured by Mettler Toledo GmbH (Greifensee, Switzerland). In this case the pH value of the samples did not change, it was done only for the confirmation of the stability of the optimized suspensions.

### 2.3. Sintering of Monolithic Alumina and Composite Alumina-Zirconia Ceramics

Monolithic Al_2_O_3_ and composite Al_2_O_3_-ZrO_2_ ceramics were prepared by conventional sintering of green bodies formed by slip casting forming method. Therefore, after the apparent viscosity measurement the suspensions were poured into previously prepared gypsum molds and air-dried. The gypsum mold draws water from the poured slip and gives a form to the green body.

**Figure 3:**
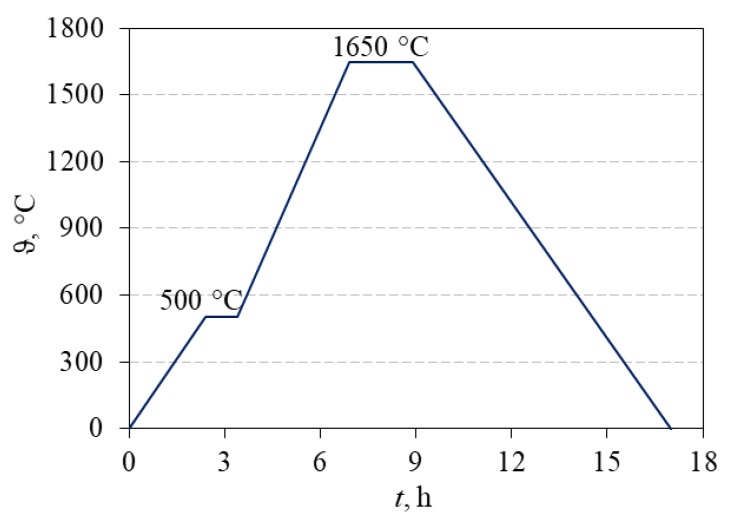
Schema of sintering regime of monolithic Al_2_O_3_ and Al_2_O_3_–ZrO_2_ composite ceramics.

Afterwards, dried samples were removed from molds. The green bodies were sintered in the high-temperature furnace P 310 (Nabertherm, Lilienthal, Germany) by the following regime: initial heating at a rate of 3 °C/min up to the temperature of 500 °C, holding at 500 °C for 1 h, followed by heating at a rate of 5 °C/min up to the temperature of 1650 °C, holding at 1650 °C for 2 h and finally slow cooling in the furnace to room temperature (Figure 3). After sintering, the Archimedes density was measured for all samples.

### 2.4. Characterisation of Monolithic Alumina and Composite Alumina-Zirconia Ceramics

Sintered samples were prepared for the following tests according to the standard ceramographic technique [28]. Surface morphology of the sintered ceramic samples was determined by the scanning electron microscope (SEM), Tescan Vega Easy Probe 3, Brno-Kohoutovice, Czech Republic operating at 10 kV, additionally equipped with energy dispersive spectrometer (EDS), Oxford Instruments, Abingdon, UK. Distribution of the elements aluminum (Al), zirconium (Zr) oxygen (O) and yttrium (Y) on fracture surface of sintered samples was determined by EDS mapping.

**Figure 4:**
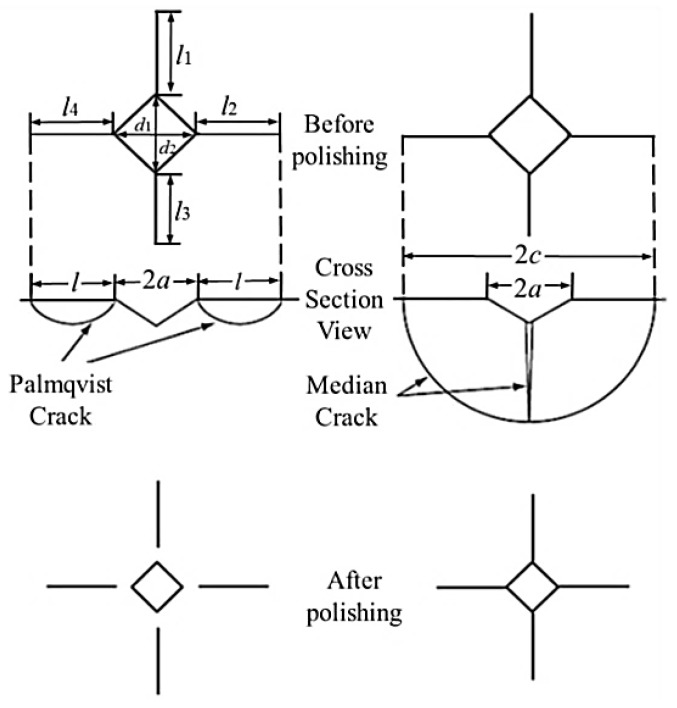
Palmqvist and median crack system developed from the Vickers indents, before and after polishing [29].

Vickers hardness (*HV*30) of sintered ceramic samples was measured under 294 N indentation load by means of hardness tester 5030 TKV (Indentec Hardness Testing Machines Ltd., West Midlands, UK). The “Vickers indentation fracture, (VIF)” or “Vickers indentation crack length” method was used for fracture toughness determination of all ceramic samples. This method uses a Vickers indenter to make a hardness indentation on a polished ceramic sample surface. The indenter creates a plastically-deformed region underneath the indenter as well as cracks that emanate radially outward and downward from the vertices of the Vickers indentation. Besides radial-median cracks, Palmqvist cracks can also occur (Figure 4). A simple way to differentiate between the two types is to polish the surface layers away: the median crack system will always remain connected to the inverted pyramid of the indentation, while the Palmqvist cracks will become detached, as shown in Figure 4.

In the order to calculate Vickers indentation fracture toughness, the lengths of these cracks were measured. Fracture toughness is calculated on the basis of the crack lengths, the indentation load, the hardness, the elastic modulus, the indentation diagonal size, and an empirical fitting constant. Nine equations based on the Palmqvist, radial-median cracks and both were found to be applicable for the fracture toughness determination of the ceramics (Table 4). The brittleness index (*B*_i_, μm^−1/2^) was calculated from the ratio of Vickers hardness and Vickers indentation fracture toughness.

## 3. Results and Discussion

**Figure 5:**
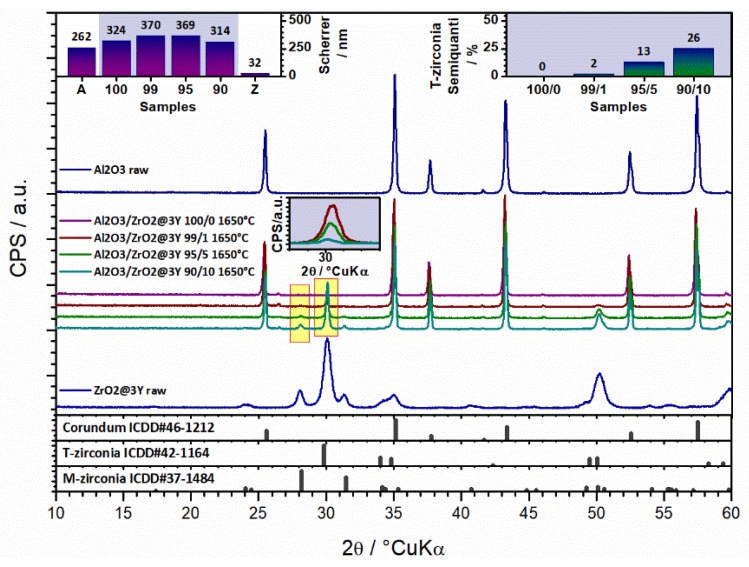
XRD patterns of raw Al_2_O_3_, ZrO_2_ and thermal treated (1650 °C) Al_2_O_3_-ZrO_2_ composite powders.

From the diffractogram in Figure 5, the qualitative crystalline composition determination was possible for all samples. The diffractogram in Figure 5 indicates that the raw Al_2_O_3_ consists of only α-Al_2_O_3_ crystalline phase (corundum) (ICDD PDF#46-1212). On the other hand, for raw ZrO_2_ powder two phases were assigned to the main phase; zirconia tetragonal phase (*t*-ZrO_2_) (ICDD PDF#42-1164) and the minor phase; zirconia monoclinic phase (*m*-ZrO_2_) (ICDD PDF#37-1484). Amorphous phase or residuals are not present. Qualitatively, sintered zirconia-toughened alumina composite shows the presence of α-Al_2_O_3_ (ICDD PDF#46-1212) as the main phase, *t*-ZrO_2_ (ICDD PDF#42-1164) as the minor phase and *m*-ZrO_2_ (ICDD PDF#37-1484) in traces. With increase of the zirconia content, both *t*-ZrO_2_ and *m*-ZrO_2_ intensity increased (the intensity increase of the *t*-ZrO_2_ phase peak is shown in Inset). Semiquantitatively, the loading of the *t*-ZrO_2_ is also shown in Inset (having in mind Al_2_O_3_ and ZrO_2_ show different absorption coefficients, the loading should only point out to a trend between different values and not to exact values).

The intensity of the *t*-ZrO_2_ strongest peak and *m*-ZrO_2_ strongest peak were compared to allow an insight in the mutual dependence of the zirconia phases (*t*-ZrO_2_ to *m*-ZrO_2_ ratio) as a function of the zirconia loading. With the introduction of 1 wt. % of zirconia, the relative content of the *m*-ZrO_2_ is about 15 wt. % (85 wt. % *t*-ZrO_2_). However, the calculation of the ratio of phases that are present in levels of about 1 % is questionable. Upon an increase in the zirconia loading to 5 wt. %, the relative presence of the *m*-ZrO_2_ is reduced to about 7 % (93 % *t*-ZrO_2_), and the similar ratio remains for further increase of the zirconia loading up to 10 wt. %. Basically, the ratio between *m*-ZrO_2_ and *t*-ZrO_2_ zirconia phases remains the same. This actually makes sense as there is no clear reason why the increased zirconia content would affect the ratio between *m*-ZrO_2_ and *t*-ZrO_2_ zirconia. The only reason could be the consequence of the phase transformation from tetragonal into monoclinic phase in the cracking zone (closure of the crack due to the volume changes). However, such microeffects are not statistically observable using a method like XRD. The important issue is that for the healing of the crack there is plenty of the main zirconia phase, the *t*-ZrO_2_ available [36,37]. As the microstructure is considered, the use of raw α-alumina yields crystallites of about 262 nm in size, while raw zirconia yields crystallites of about 32 nm. Crystallite size was calculated by applying the Scherrer equation on the XRD patterns. Some growth occurs in the subsequent process, as ceramic with pure alumina yields 324 nm, where 1 and 5 wt. % of zirconia in composites marginally affect the crystallite size (370 and 369 nm). 10 wt. % of zirconia definitively impacts the microstructure of composites, reducing the crystallite size to 314 nm.

**Figure 6:**
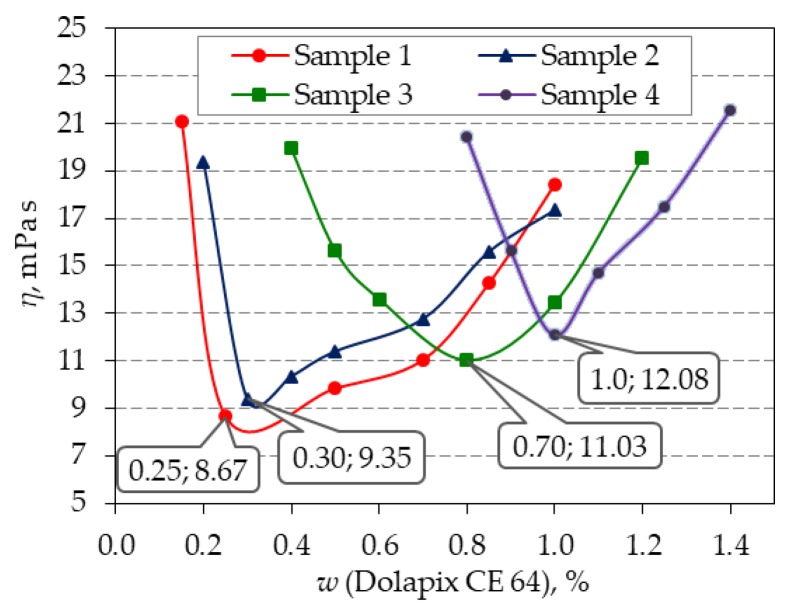
Influence of amounts of Dolapix CE 64 dispersant (wt. %) on apparent viscosity (*η*) of the 70 wt. % suspensions with ceramic powder composition of sample 1: 100 wt. % Al_2_O_3_; sample 2: 99 wt. % Al_2_O_3_ + 1 wt. % ZrO_2_; sample 3: 95 wt. % Al_2_O_3_ + 5 wt. % ZrO_2_; sample 4: 90 wt. % Al_2_O_3_ + 10 wt. % ZrO_2_.

Viscosity measurements were used for the suspension stability estimation. Rheological measurements showed that measured apparent viscosity increases with the increasing zirconia content. The optimal amount of Dolapix CE 64 also increases with the increasing zirconia content. The diagram in Figure 6 shows the apparent viscosity at the shear rate of app. 50 s^−1^, which is the shear rate of the gravity slip casting.

The obtained minimum viscosity values represent the most stable suspension, which served as the guideline for the preparation of the most suitable ceramic suspensions to be poured into the mold to prepare green bodies and to be sintered, in order to get the samples of monolithic Al_2_O_3_ and Al_2_O_3_-ZrO_2_ composite ceramics.

By comparing the results in Figure 6 it is evident that the optimum amount of the dispersant increases with increased ZrO_2_ content. The reason for this is probably the fact that this component has finer particles. Ceramic particles of smaller dimensions have a larger specific surface. Therefore, the area that the macromolecules of the dispersant must cover to ensure the stability of the system is larger. Hence, a higher amount of the dispersant is needed to stabilize such suspensions [18].

Table 3 shows the composition of the tested suspensions, while the optimal compositions (i.e., optimal dispersant amount) are shown in Table 5.

In this study no typical sedimentation tests were performed [14,38], meaning, the sedimentation rate of the suspension was not observed depending on the pH value. Only the sedimentation rates for suspensions with the optimum proportion of the dispersant at their "natural" pH value were measured. The measured pH values of the real samples and their mean values of three measurements are shown in Table 6. 

The prepared suspensions did not show any indication of phase separation after 3 days and the suspensions were still homogeneous. Full separation of the solid and liquid phase was observed after more than seven days in all four suspension groups. For this reason, the prepared suspensions can be considered stable [14,38], without the need for pH control.

**Figure 7:**
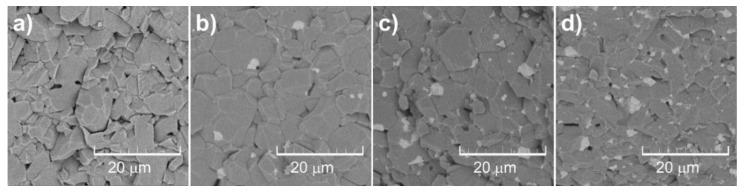
Surface fracture of Al_2_O_3_–ZrO_2_ composite ceramics with different content of ZrO_2_: (**a**) 0 wt. %; (**b**) 1 wt. %; (**c**) 5 wt. %; (**d**) 10 wt. % prepared by slip casting and sintering at 1650 °C.

**Figure 8:**
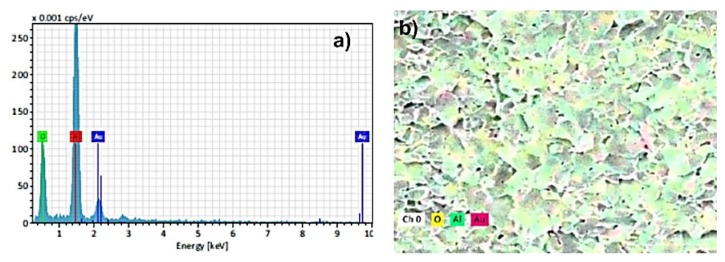
(**a**) EDS spectra and (**b**) SEM micrographs of surface fracture of Al_2_O_3_ ceramics.

**Figure 9:**
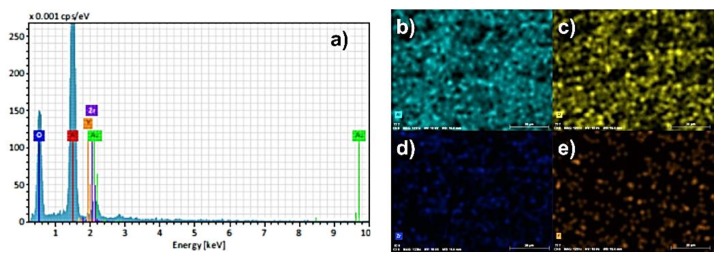
(**a**) EDS spectra of surface fracture of Al_2_O_3_–ZrO_2_ ceramics with 1 wt. % of ZrO_2_ and corresponding element mappings: (**b**) Aluminum (Al); (**c**) Oxygen (O); (**d**) Zirconium (Zr); (**e**) Yttrium (Y).

**Figure 10:**
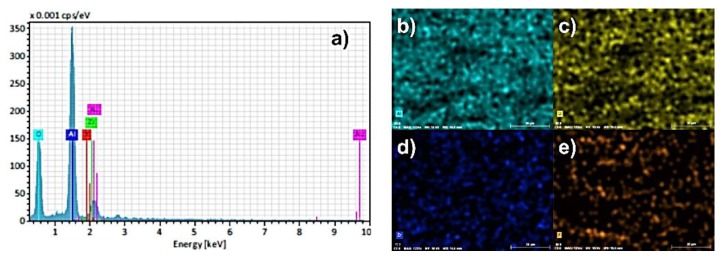
(**a**) EDS spectra of surface fracture of Al_2_O_3_–ZrO_2_ ceramics with 5 wt. % of ZrO_2_ and corresponding element mappings: (**b**) Aluminum (Al); (**c**) Oxygen (O); (**d**) Zirconium (Zr); (**e**) Yttrium (Y).

**Figure 11:**
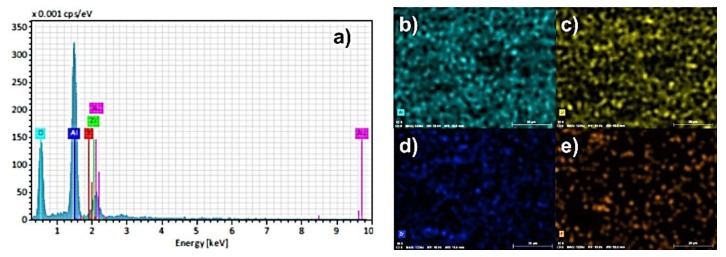
(**a**) EDS spectra of surface fracture of Al_2_O_3_–ZrO_2_ ceramics with 10 wt. % of ZrO_2_ and corresponding element mappings: (**b**) Aluminum (Al); (**c**) Oxygen (O); (**d**) Zirconium (Zr); (**e**) Yttrium (Y).

The results of SEM-EDS analysis of surface fracture of sintered monolithic Al_2_O_3_ and Al_2_O_3_–ZrO_2_ composite ceramics are shown in Figure 7, Figure 8, Figure 9, Figure 10 and Figure 11. It can be seen (Figure 7b–d, Figure 9, Figure 10 and Figure 11) that the ZrO_2_ particles (the brighter phase) are distributed in Al_2_O_3_ matrix, with some agglomerates of ZrO_2_ and some pores. These observations were additionally confirmed by the SEM-EDS mapping of the surface fracture of sintered samples (Figure 9, Figure 10 and Figure 11).

The composites with 5 and 10 wt. % ZrO_2_ have shown larger agglomerates of ZrO_2_ in Al_2_O_3_ matrix compared to 1 wt. % ZrO_2_ (Figure 7b–d)). The pores are mostly distributed around ZrO_2_ agglomerates. Also, the increasing of ZrO_2_ content has resulted in a reduction of Al_2_O_3_ grain size. All these microstructural characteristics typically affect the mechanical properties, such as hardness and fracture toughness.

The results of measuring the density of sintered samples (Al_2_O_3_ and Al_2_O_3_-ZrO_2_ composite) confirm that the density increases with increasing the ZrO_2_ content (Table 7). The theoretical density of pure Al_2_O_3_ is 3.97 g/cm^3^ and of pure ZrO_2_ 6.10 g/cm^3^. From these theoretical densities of pure ceramics, weight content of components, and from the measured bulk densities, the relative densities of each sample were calculated (Table 7). The highest relative densities were recorded for pure Al_2_O_3_ and for the composite with 1 wt. % ZrO_2_. When ZrO_2_ content was increased to 5 and 10 wt. %, the relative density decreased and consequently the porosity has increased. Similar results were published previously [39].

The Vickers indentation method was used for the determination of hardness (*HV*30), fracture toughness (*K*_IC_) and brittleness index (*B*_i_) of sintered samples of monolithic Al_2_O_3_ and Al_2_O_3_-ZrO_2_ composite ceramics. The results (Table 7) showed that the hardness of the prepared samples decreased with the increased ZrO_2_ content. There are several reasons for this phenomenon. One of them is the fact that zirconia has lower hardness than Al_2_O_3_. Also, as previously described, the microstructure analysis of composites has shown the coarsening of ZrO_2_ grains and consequently the formation of porosity. These findings are in correlation with other publications [2,40,41]. Higher relative density, hence higher hardness, may be achieved when, for example, hot-pressing (HP) is used to prepare Al_2_O_3_-ZrO_2_ composites [42], hot isostatic pressing [43] or by spark plasma sintering (SPS) [44].

**Figure 12:**
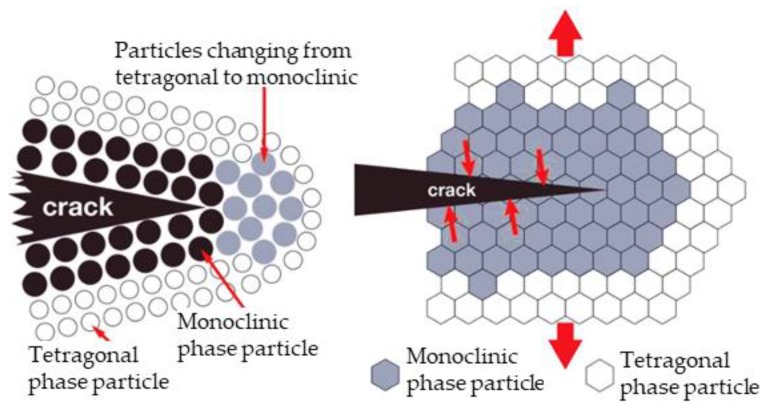
Transformation toughening mechanism in zirconia [29].

The fracture toughness of the tested samples (Table 8) also increases with the increase of the ZrO_2_ amount for all applied mathematical models. These results may be assigned to possible phase transformations, formation of microcracks or crack branching [2]. Composite ceramics with ZrO_2_ grains, exhibit the martensitic phase transformation: when the stress is applied, tetragonal ZrO_2_ grains transform into monoclinic ZrO_2_ at the crack tip (Figure 12). 

This transformation induces a volume expansion from 3–5 %, which prevents the crack propagation due to induced compressive stress. This phenomenon is known as the transformation toughening. The larger the ZrO_2_ content in the composite ceramics, the higher the possibility of crack closure [29]. Besides the zirconia phase transformation, it was found that the alumina grain bridging is also a toughening mechanism which contributes to an increase in toughness of similar alumina zirconia composites [30].

The ratio of the Vickers crack length and half of the Vickers indentation diagonal (*c/a*, Table 7) indicates the crack type and the crack depth. The crack type is an indirect indicator of material toughness [1,29,35,45]. According to the obtained results (Table 7) and interpretation from literature sources [1,29,35,45], median cracks occurred in the first three groups of samples, while shallow, Palmqvist cracks appeared in the fourth group (90 wt. % Al_2_O_3_ + 10 wt. % ZrO_2_). It should be emphasized that the *c/a* ratio for the first three groups of samples is slightly above the limit value of 2.5, so it can be concluded that even low ZrO_2_ content reduces the crack depth, i.e., increases the toughness. According to Tang et al. [45], samples with a *c/a* ratio between 2.5 and 3.5 indicate the presence of both types of cracks (both Palmqvist and median), therefore represent the transition between the two types of cracks. According to this interpretation, it is possible to determine the presence of the transitional crack shape (between median and Palmqvist type) in the first three groups of samples, while in the fourth sample group only the Palmqvist cracks occur. According to the obtained *c/a* values, from 2.3 to 3.9, the most adequate model for the indentation fracture toughness is the Langford model, since it is applicable to both crack types (median and Palmquist).

**Figure 13:**
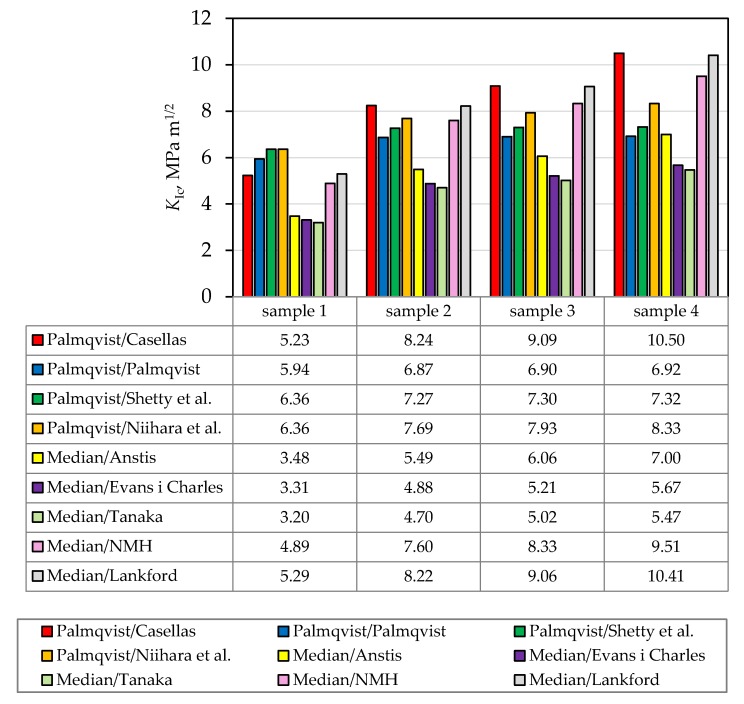
Comparison of Vickers indentation fracture toughness values for different crack types, models and all samples (NMH-Niihara, Morena and Hasselman).

Since not all groups of samples developed the same crack type, not all selected models were applicable to all four sample groups [32]. Nevertheless, all models showed the same trend, i.e., an increase in fracture toughness with increasing ZrO_2_ content (Table 8 and Figure 13). The obtained results show that the toughness of monolithic Al_2_O_3_ ceramics can be improved by the addition of ZrO_2_ nanoparticles.

For each sample, the brittleness index (*B*_i_, μm^−1/2^) was calculated from the ratio of values of Vickers hardness (*HV*30, GPa) and the Vickers indentation fracture toughness (*K*_IC_, MPa m^1/2^) using Equation (1) [46]:

(1) $$B_{i}=\frac{HV}{K_{\mathrm{IC}}}$$

**Figure 14:**
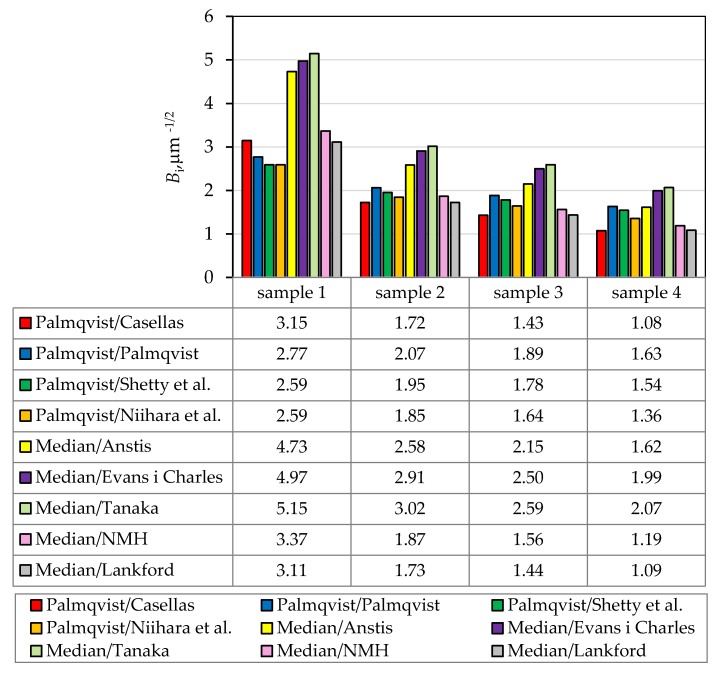
Comparison of brittleness index (*B*_i_, μm^−1/2^) values for different crack types, models and all samples (NMH-Niihara, Morena and Hasselman).

Calculated values of the brittleness index for all sintered samples are shown in Figure 14. All models showed that the brittleness index decreases with increasing ZrO_2_ content.

## 4. Conclusions

In this paper, monolithic Al_2_O_3_ and composite Al_2_O_3_–ZrO_2_ ceramics samples were formed by slip casting stable suspensions in gypsum molds. The following conclusions can be drawn as a result of the research:

- XRD analysis confirmed that alumina powder consists of α-phase alumina (corundum). The XRD results confirm the changing levels of alumina and zirconia in the composites, and point out to the stable ratio between main tetragonal phase and monoclinic zirconia phase in traces. In addition, the change of the crystallite sizes because of the zirconia loading was quantified.
- With the addition of the commercial dispersant DOLAPIX CE 64, it is possible to prepare stable 70 wt. % aqueous suspensions of monolithic Al_2_O_3_ and composite Al_2_O_3_-ZrO_2_ ceramics using commercial powders.
- 0.25 wt. % of DOLAPIX CE 64 dispersant is required to stabilize the 70 wt. % aqueous suspensions of monolithic Al_2_O_3_ ceramics.
- 0.3 wt. % of DOLAPIX CE 64 dispersant is required to stabilize the 70 wt. % aqueous suspensions of composite Al_2_O_3_–ZrO_2_ ceramics, composed of 99 wt. % Al_2_O_3_ and 1 wt. % ZrO_2_.
- 0.7 wt. % of DOLAPIX CE 64 dispersant is required to stabilize the 70 wt. % aqueous suspensions of composite Al_2_O_3_–ZrO_2_ ceramics, composed of 95 wt. % Al_2_O_3_ and 5 wt. % ZrO_2_
- 1 wt. % of DOLAPIX CE 64 dispersant is required to stabilize 70 wt. % aqueous suspensions of composite Al_2_O_3_–ZrO_2_ ceramics, composed of 90 wt.% Al_2_O_3_ and 10 wt.% ZrO_2_.
- Apparent viscosity and the required amount of Dolapix CE 64 increase with increasing the zirconia content.
- Green bodies of monolithic Al_2_O_3_ and composite Al_2_O_3_-ZrO_2_ ceramics were formed by slip casting process in plaster molds. After drying, the green bodies were sintered at a temperature of 1650 °C.
- SEM-EDS analysis of prepared composite ceramics showed that ZrO_2_ particles are dispersed in Al_2_O_3_ matrix with some agglomerates of ZrO_2_, and pores.
- The obtained *c/a* values ranging from 2.3 to 3.9 indicate that the Langford model is the most appropriate model for the indentation fracture toughness, because this model can be applied to both median and Palmquist crack types.
- By adding ZrO_2_ nanoparticles in alumina matrix, the hardness has decreased because the hardness of tetragonal zirconia is lower than alumina. Also, the addition of ZrO_2_ nanoparticles has caused an increase in total porosity, hence lowering the hardness.
- On the other hand, the fracture toughness of alumina matrix has increased by adding ZrO_2_ nanoparticles as a result of the synergistic effect of transformation toughening and the microstructural changes.

## Figures and Tables

**Table 1:** Chemical composition of Al_2_O_3_ powder.

Component	MgO	Fe_2_O_3_	SiO_2_	Na_2_O	CaO	Al_2_O_3_
wt. %	0.066	0.015	0.02	0.05	0.013	balance

**Table 2:** Chemical composition of the ZrO_2_ powder.

Component	Y_2_O_3_	ZrO_2_
mol. %	3	97

**Table 3:** The composition of suspensions for determining the optimal amount of dispersant DOLAPIX CE 64.

Sample	wt. (Ceramic Powder), %	Ceramic Powder Composition	wt. (MgAl_2_O_4_) *, %	wt. (Dolapix CE 64) *, %
1	70	100 wt. % Al_2_O_3_	0.2	0.15 to 1.0
2	70	99 wt. % Al_2_O_3_ + 1 wt. % ZrO_2_	0.2	0.2 to 1.0
3	70	95 wt. % Al_2_O_3_ + 5 wt. % ZrO_2_	0.2	0.4 to 1.2
4	70	90 wt. % Al_2_O_3_ + 10 wt. % ZrO_2_	0.2	0.8 to 1.4

* weight percentage based on the amount of dry ceramic powder.

**Table 4:** Models by different authors for calculation of Vickers indentation fracture toughness (*K*_IC_) values for different crack types [29].

Crack Type	Model	Author(s) of Model
Palmqvist	$K_{\mathrm{IC}}=0.024\times \frac{F}{c^{1.5}}\times {\left(\frac{E}{HV}\right)}^{0.5}$	Casellas [2,30]
Palmqvist	$K_{\mathrm{IC}}=0.0028\times HV^{0.5}{\left(\frac{F}{T}\right)}^{0.5}$	Palmqvist [31]
Palmqvist	$K_{\mathrm{IC}}=0.0319\times \frac{F}{a\cdot l^{0.5}}$	Shetty et al. [32]
Palmqvist	$K_{\mathrm{IC}}=0.0089\times {\left(\frac{E}{HV}\right)}^{0.4}\times \frac{F}{a\cdot l^{0.5}}$ for 0.25 < *l*/*a* < 2.5	Niihara et al. [32]
Median	$K_{\mathrm{IC}}=0.016\times \frac{F}{c^{1.5}}\times {\left(\frac{E}{HV}\right)}^{0.5}$	Anstis [1,2,33]
Median	$K_{\mathrm{IC}}=0.0752\times \frac{F}{c^{1.5}}$	Evans and Charles [34]
Median	$K_{\mathrm{IC}}=0.0725\times \frac{F}{c^{1.5}}$	Tanaka [34]
Median	$K_{\mathrm{IC}}=0.0309\times {\left(\frac{E}{HV}\right)}^{0.4}\times \frac{F}{c^{1.5}}$	Niihara, Morena and Hasselman (NMH) [35]
Any kind	$K_{\mathrm{Ic}}=0.0782\times \left(HV\times a^{0.5}\right){\left(\frac{E}{HV}\right)}^{0.4}\times {\left(\frac{c}{a}\right)}^{-1.56}$	Lankford [32]

*F*, applied load during Vickers test (N); *c*, the crack length from the center of the indentation to the crack tip (m); *E*, Young’s modulus (GPa); *HV*, the Vickers hardness (GPa); *l*, the crack length measured from vertices of the indentation to the crack tip (m); *T*, the total crack length (m): *T* = *l*_1_ + *l*_2_ + *l*_3_ + *l*_4_; *a*, half of the indentation diagonal (m).

**Table 5:** The compositions of stable suspensions for preparation of monolithic Al_2_O_3_ and Al_2_O_3_–ZrO_2_ composite ceramics.

Sample	wt. (Ceramic Powder), %	Ceramic Powder Composition	wt. (MgAl_2_O_4_) *, %	Optimal Amount of Dispersant *
1	70	100 wt. % Al_2_O_3_	0.2	0.25 wt. %
2	70	99 wt. % Al_2_O_3_ + 1 wt. % ZrO_2_	0.2	0.30 wt. %
3	70	95 wt. % Al_2_O_3_ + 5 wt. % ZrO_2_	0.2	0.70 wt. %
4	70	90 wt. % Al_2_O_3_ + 10 wt. % ZrO_2_	0.2	1.00 wt. %

* wt., weight percent based on the applied ceramic dry powder.

**Table 6:** Measurement results of pH values on real samples of stable suspensions of monolithic Al_2_O_3_ and Al_2_O_3_-ZrO_2_ composite ceramics ($\bar{x}$-mean, *s*-experimental standard deviation).

pH Value	Sample			
	100 wt. % Al_2_O_3_	99 wt. % Al_2_O_3_ + 1 wt. % ZrO_2_	95 wt. % Al_2_O_3_ + 5 wt. % ZrO_2_	90 wt. % Al_2_O_3_ + 10 wt. % ZrO_2_
$\bar{x}$ ± *s*	8.92 ± 0.06	8.36 ± 0.05	8.52 ± 0.09	8.26 ± 0.10

**Table 7:** The density, porosity and hardness of Al_2_O_3_ and Al_2_O_3_-ZrO_2_ samples and the *c/a* ratio. Where, *c* is the crack length from the center of the indentation to the crack tip in m and *a* is a half of the indentation diagonal.

Sample	Composition	Bulk Density, g/cm^3^	Relative Density, %	Total Porosity, %	*HV*30	*c*/*a*
1	100 wt. % Al_2_O_3_	3.882	98.04	1.96	1679	3.89
2	99 wt. % Al_2_O_3_ + 1 wt. % ZrO_2_	3.920	98.21	1.79	1447	2.82
3	95 wt. % Al_2_O_3_ + 5 wt. % ZrO_2_	3.931	96.43	3.57	1328	2.59
4	90 wt. % Al_2_O_3_ + 10 wt. % ZrO_2_	3.938	94.14	5.86	1153	2.28

**Table 8:** Values of the Vickers indentation fracture toughness for different crack type and models.

Crack Type	Author(s) of Model	*K*_IC_, MPa m^1/2^			
		Sample 1 (100 wt. % Al_2_O_3_)	Sample 2 (99 wt. % Al_2_O_3_ + 1 wt. % ZrO_2_)	Sample 3 (95 wt. % Al_2_O_3_ + 5 wt. % ZrO_2_)	Sample 4 (90 wt. % Al_2_O_3_ + 10 wt. % ZrO_2_)
Palmqvist	Casellas [2,30]	5.23	8.24	9.09	10.50
Palmqvist	Palmqvist [31]	5.94	6.87	6.90	6.92
Palmqvist	Shetty et al. [32]	6.36	7.27	7.30	7.32
Palmqvist	Niihara et al. [32]	6.36	7.69	7.93	8.33
Median	Anstis [1,2,33]	3.48	5.49	6.06	7.00
Median	Evans and Charles [34]	3.31	4.88	5.21	5.67
Median	Tanaka [34]	3.20	4.70	5.02	5.47
Median	Niihara, Morena and Hasselman (NMH) [35]	4.89	7.60	8.33	9.51
Any kind	Lankford [32]	5.29	8.22	9.06	10.41
